# Spontaneous recovery in Autistic Spectrum Disorders ‐ A myth?

**DOI:** 10.4103/0019-5545.64581

**Published:** 2010

**Authors:** M. N. Helal, I. Mushtaq, S. Sankar

**Affiliations:** Leicester CAHMS, Westcotes House, Westcotes Drive, Leicester, LE30QU, U.K; 1 Northampton CAHMS, 8-Notre Dame Mews, Northampton NN1 2 BG U.K

Sir,

Autism is not a disease. There is no blood test, neuroimaging or electroencephalography (EEG) test to diagnose or confirm autism.[1] The coordinated efforts of a team of different professionals, including a pediatrician, child psychiatrist, speech therapist, clinical/educational psychologist and specialist school teacher, along with parents help reach a conclusion based on all available information rather than absolute criteria.

In western countries, usually pediatricians take the lead in coordinating the assessment with the help of a multiprofessional team, and unsurprisingly are not aware of International Statistical Classification of Diseases and Related Health Problems 10th Revision (ICD-10) and Diagnostic and Statistical Manual of Mental Disorders, 4th Edition (DSM-IV) criteria.

However, in this case report[2] it seems that the diagnosis was made only by psychiatrists, providing limited grounds for diagnosis. We would like to raise a few points here. They argue that if the diagnostic criteria for pervasive developmental disorders (PDD) were met earlier (not later), the patient may be called greatly recovered or one may be questioning the initial diagnosis. The authors have used Childhood Autism Rating Scale (CARS) for diagnosis, which itself has its weaknesses in diagnosing autism. One can criticize the CARS because it does not separate ‘normal’ and ‘mildly affected’. These borderline cases overlap with cognitive impairment, obsessive-compulsive disorder, semantic-pragmatic language disorder without autism, and other co-morbid phenotypes.[3] Another weakness of the CARS is its unreliable discrimination of young children with autism from mental-age matched children with other disorders, especially limited language.[1]

Research on autism has shown that three-quarters have intelligence quotient (IQ) score in the ‘retarded’ range and this finding appears to represent true intellectual impairment; the cognitive defects remain there and there is no improvement in IQ.[4] However, the case report mentions that P’s somatic quotient / development quotient (SQ/DQ) was 25-30, which later improved to 40-45. This makes it difficult to understand whether he actually had autism with mental retardation; or mental retardation alone or neither of them.

An area of interest for further research for clinicians would be to devise clinical instruments which could differentiate between autism and other mental disorders with similar symptoms.
